# Toughening of Poly(L-lactide) with Blends of Poly(*ε*-caprolactone-*co*-L-lactide) in the Presence of Chain Extender

**DOI:** 10.1155/2018/1294397

**Published:** 2018-09-12

**Authors:** Yaowalak Srisuwan, Yodthong Baimark, Supakij Suttiruengwong

**Affiliations:** ^1^Biodegradable Polymers Research Unit, Department of Chemistry and Center of Excellence for Innovation in Chemistry, Faculty of Science, Mahasarakham University, Mahasarakham 44150, Thailand; ^2^Department of Materials Science and Engineering, Faculty of Engineering and Industrial Technology, Silpakorn University, Sanamchandra Palace Campus, Nakhon Pathom 73000, Thailand

## Abstract

A poly(*ε*-caprolactone-*co*-L-lactide) copolyester was synthesized and employed to toughen poly(L-lactide) (PLLA) by reactive melt blending in the presence of an epoxy-based chain extender. The effects of chain extension reaction and copolyester content on properties of PLLA-based blends were studied. The chain extension reaction reduced crystallinity and melt flow index of PLLA/copolyester blends. Meanwhile the copolyester blending improved the crystallinities of the chain-extended PLLA up to 20 wt% copolyester. The phase compatibility between PLLA matrix and dispersed copolyester phases was enhanced by the chain extension reaction. The impact strength of chain-extended PLLA increased with the contents of copolyester and chain extender.

## 1. Introduction

Poly(L-lactic acid) or poly(L-lactide) (PLLA) is one of the most well-known biodegradable polymers that has attracted increasing interest for use in clinical applications such as drug delivery systems, tissue engineering, and long-term implantable devices [1–4]. This is due to its low toxicity, biocompatibility, biodegradability, and processability [5–7]. However, inherent brittleness of PLLA limits its practical applications when toughness is desired [7, 8].

Toughness of PLLA has been improved either by plasticization, copolymerization, or melt blending with a variety of flexible polymers or rubbers [8, 9]. For plasticizer addition, the migration of plasticizers from the matrix to surface of PLLA is still the main problem [10, 11]. Melt blending is a much more convenient and economical method than copolymerization. For this purpose, various biodegradable polymers such as poly(*ε*-caprolactone) [12], poly(butylene succinate) [13], polyhydroxyalkanoate copolymer [14], poly(butylene adipate-*co*-terephthalate) [15], and poly(*ε*-caprolactone-*co*-D,L-lactide) [16] have been melt blended with PLLA to enhance toughness. The compatibility and rubbery character of the dispersed phase in the PLLA matrix are important factors for improving the toughness of PLLA. The size and size distribution of the dispersed phase which depend on its compatibility should reach an optimum value in order to maximize the toughness of PLLA [16].

Fully biodegradable poly(*ε*-caprolactone-*co*-L-lactide) copolyesters [P(CL-*co*-LLA)] are more flexible polymers than PLLA due to their lower glass transition temperatures [17]. P(CL-*co*-LLA)s have been investigated as nerve guide tubes [18] and have also been used to improve the toughness of PLLA by block copolymerization [17, 19] and used as compatibilizers of PLLA/PCL blends by both solution and melt blending [20, 21]. However, melt blending of PLLA with P(CL-*co*-LLA) has been scarcely published. A high molecular weight copolyester with a *ε*-caprolactone/L-lactide (CL/LLA) ratio of 50/50 mol% is not completely amorphous due to differences in monomer reactivities. The small melting peaks of the LLA segments of the 50/50 mol% P(CL-*co*-LLA)s were still detected during storage at room temperature [17, 22]. This might reduce elasticity of the P(CL-*co*-LLA)s for PLLA toughening. It has been reported that the high molecular weight 60/40 mol% P(CL-*co*-LLA) exhibited a complete amorphous character that can improve the toughness of PLLA by block copolymerization better than the 50/50 mol% P(CL-*co*-LLA) [23]. Thus, in this work the 60/40 mol% P(CL-*co*-LLA) was chosen to blend with the PLLA.

Chain extension is a chemical reaction of polymer molecules that uses a chain extender to expand molecular size. Joncryl® are effective multifunctional chain extenders for polyesters that have epoxy groups, which can react with the carboxyl and hydroxyl end-groups of polyesters. Joncryl® have been used to control the melt flow properties and thermal-mechanical degradation of PLLA by forming long-chain branched structures [24, 25]. Joncryl® has also been used to compatibilize polyester blends such as PLLA-poly(butylene adipate-*co*-terephthalate) blends [26]. However, PLLA toughening with poly(*ε*-caprolactone-*co*-L-lactide) [P(CL-*co*-LLA)] in the presence of the chain extender has not been reported.

Therefore, in this work, a high molecular weight 60/40 mol% P(CL-*co*-LLA) was chosen for use as a biodegradable toughness enhancer for PLLA. Influences of the 60/40 mol% P(CL-*co*-LLA) blend ratio and content of chain extender on the thermal transitions, phase compatibility, and mechanical properties of the PLLA-based blends could be established.

## 2. Materials and Methods

### 2.1. Materials

The L-lactide (LLA) monomer was prepared using polycondensation followed by thermal depolymerization from L-lactic acid (88%, Purac, Thailand). The LLA monomer was four times purified by recrystallization from ethyl acetate before drying in a vacuum oven at 55°C for 24 h. The *ε*-caprolactone (CL) monomer (99%, Acros Organics, USA) and 1-dodecanol (98%, Fluka, Switzerland) were purified by distillation under reduced pressure before use. Stannous octoate [Sn(Oct)_2_], (95%, Sigma, USA), was used without further purification. All reagents used were of analytical grade. A styrene-acrylic multifunctional epoxide oligomeric agent (Joncryl® ADR 4368, BASF, Thailand) in flake form with a molecular weight of 6,800 g/mol (an epoxy equivalent weight of 285 g/mol) was used as an epoxy-based chain extender.

### 2.2. Synthesis of PLLA

PLLA was synthesized by ring-opening polymerization in bulk from the LLA monomer, at 165°C for 2.5 h, under a nitrogen atmosphere using Sn(Oct)_2_ (0.01 mol%) and 1-dodecanol (0.14 mol%) as the initiating system. The obtained PLLA was granulated before drying in a vacuum oven at 110°C for 3 h to remove the unreacted LLA monomer. The number-averaged molecular weight (M_n_) and dispersity index (DI) values of the PLLA obtained from the gel permeation chromatography (GPC, Waters e2695 separation module) in tetrahydrofuran at 40°C were 88,400 g/mol and 2.3, respectively. The L-enantiomer content obtained from the ADP220 Bellingham and Stanley Polarimeter in chloroform at 25°C was 96%.

### 2.3. Synthesis of Copolyester

**Figure 1:**
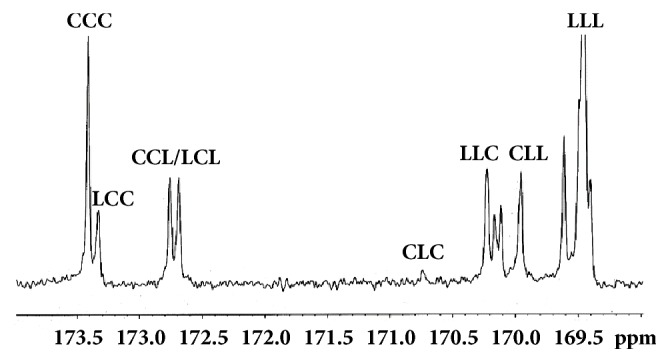
Expanded carbonyl regions of the ^13^C-NMR sceptrum of copolyester (peak-triad assignments as shown).

The poly(*ε*-caprolactone-*co*-L-lactide) random copolyester with CL/LLA ratio of 60/40 mol% was synthesized by ring-opening polymerization in bulk at 145°C for 12 h under a nitrogen atmosphere using Sn(Oct)_2_ (0.02 mol%) and 1-dodecanol (0.12 mol%) as the initiating system. The resulting copolyester was cut into small pieces before drying in a vacuum oven at 110°C for 3 h to remove the unreacted monomers. The M_n_ amd DI values of the copolyester obtained from GPC were 85,000 g/mol and 2.1, respectively. The copolyester was completely amorphous. The CL/LLA ratio and glass transition temperature (T_g_) of the copolyester obtained from the 300 MHz Bruker DPX300 ^1^H-NMR and differential scanning calorimetry (DSC) methods were 58/42 mol% and -25°C, respectively. Average block lengths of the LLA (*l*_LL_) and CL (*l*_C_) units from 75 MHz Bruker DPX300 ^13^C-NMR spectrum were calculated from triad peak intensities (*I*) by (1) and (2), respectively [17, 19]. The various triad peaks referred to in these two equations are labelled in Figure 1. For NMR analysis, deuterated chloroform was used as a solvent.

(1) lLL=12ILLL+ILLC+ICLL/2ILLC+ICLL/2+ICLC+1

(2) lC=ICCC+ILCCICCL+ILCL+1

The subscripts “LL” and “C” in (1) and (2) were LLA and CL repeating units, respectively. There were 3.0 and 2.4 for the *l*_LL_ and *l*_C_, respectively.

### 2.4. Preparation of PLLA/Copolyester Blends

The PLLA, copolyester, and Joncryl® chain extender were dried in a vacuum oven at 50°C overnight before melt blending. The 60/40 (w/w) PLLA/copolyester mixtures with (1.0 and 2.0 phr Joncryl®) and without Joncryl® were* in situ* melt blended to prepare PLLA/copolyester blends using a HAAKE Polylab OS Rheomix batch mixer at 190°C for 5 min with a rotor speed of 100 rpm. Chain-extended blends with PLLA/copolyester blend ratios of 100/0, 90/10, 80/20, and 60/40 (w/w) were also prepared by the same method using 2.0 phr Joncryl® content. The obtained PLLA/copolyester blends were granulated to obtain blend pellets and dried in a vacuum oven at 50°C overnight before characterization and compression molding.

The compressed specimens of PLLA/copolyester blends were prepared for mechanical testing using a Carver Auto CH laboratory press at 190°C without compression force for 2 min and with a 5-ton compression force for 4 min. The compressed specimens were kept at room temperature for 24 h before characterization of mechanical properties.

### 2.5. Characterization of PLLA/Copolyester Blends

Thermal transitions of the blends were determined with a Perkin-Elmer Pyris Diamond differential scanning calorimeter (DSC) under nitrogen flow. For a typical experiment, 3–5 mg of each sample was heated at 200°C/min for 3 min to erase its thermal history. Then, the sample was quenched to -40°C according to the DSC instrument's own default cooling mode before heating from of -40 to 200°C. The degree of crystallinity (*X*_c_) of the PLLA was calculated from the enthalpies of melting (ΔH_m_) and cold crystallization (ΔH_cc_) using the following equation:

(3) $$\begin{array}{c} X_{c}\left(\;\%\;\right)=\left[\;\frac{\left(\Delta H_{m}-\Delta H_{cc}\right)}{\left(93\times f_{PLLA}\right)}\;\right]\times 100 \end{array}$$

where *f*_PLLA_ is the weight fraction of the PLLA in the blends, and the enthalpy of melting of PLLA of *X*_c_ = 100% was 93 J/g [27].

The melt flow index (MFI) of the blends was determined using a Tinius Olsen MP1200 melt flow indexer. The temperature was kept uniform at 190°C, and a 2.16 kg load was applied on a 100 g rod used as a plunger to extrude the molten blends. The MFI was averaged from at least five determinations.

The phase morphology of the blend film cryofractures was observed by scanning electron microscopy (SEM) using a JEOL JSM-6460LV SEM. The blend films were immersed in liquid nitrogen for 20 min before film fracture. The film samples were coated with gold to enhance conductivity before scanning.

The tensile properties, including stress at break, elongation at break, and initial Young's modulus, of the blend films were determined at 25°C and 65% relative humidity with a Lloyds LRX+ Universal Mechanical Testing Machine. The film samples (100 × 10 × 0.2 mm) were tested with a gauge length of 50 mm and a crosshead speed of 50 mm/min. The tensile properties were averaged from at least five measurements for each sample.

Notched izod impact tests of the blend specimens (65 × 13 × 3.2 mm) were measured according to ASTM D256 using a Zwick model Pendulum impact tester B5102.202. An average value of five specimens was taken for each sample.

Hardness of the blend specimens (65 × 13 × 3.2 mm) was obtained according to ASTM D2240 using a Landtek HT-6510 Shore D durometer. The average of five values determined from various sites of each specimen was estimated.

## 3. Results and Discussion

### 3.1. Thermal Transitions

**Figure 2:**
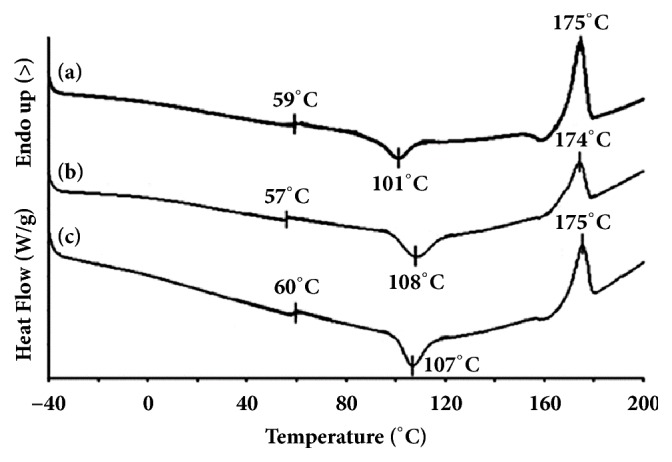
DSC curves of 60/40 (w/w) PLLA/copolyester blends: (a) without and with Joncryl® contents of (b) 1.0, and (c) 2.0 phr.

**Figure 3:**
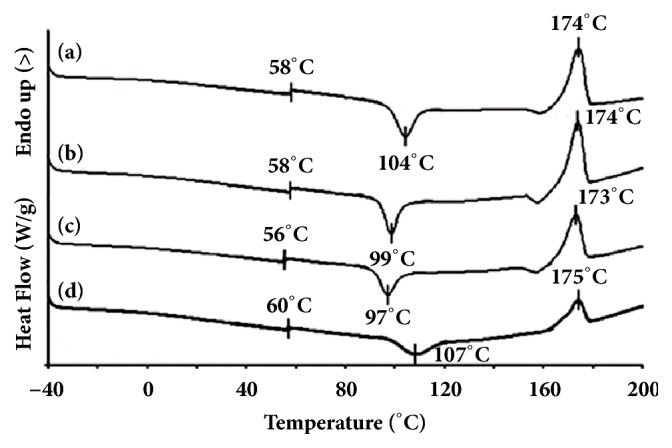
DSC curves of chain-extended blends with PLLA/copolyester ratios of (a) 100/0, (b) 90/10, (c) 80/20, and (d) 60/40 (w/w).

Thermal transitions including T_g_, cold crystallization temperature (T_cc_), and melting temperature (T_m_) of the blends were determined from the DSC curves shown in Figures 2 and 3 to investigate the influences of Joncryl® content and PLLA/copolyester blend ratio, respectively. The DSC results are summarized in Table 1.

The T_g_ and T_m_ of 60/40 (w/w) blends with different Joncryl® contents were in the ranges 57–60°C and 174–175°C, respectively, indicating that the chain extension did not affect the amorphous region of the PLLA matrix in the blends [28]. The copolyester was completely amorphous. It had no small melting peaks of LLA segments from the DSC curve when it was stored at room temperature for at least 14 days. This may be explained by the fact that the distinct values between *l*_LL_ and *l*_C_ from ^13^C-NMR were lower than the 50/50 mol% copolyester in the literatures [17, 19]. The *X*_c_ of non-chain-extended 60/40 (w/w) blend (25.1%) drastically decreased to 3.2% and 3.9% when the Joncryl® contents were 1.0 and 2.0 phr, respectively. The T_cc_ values of chain-extended blends (108 and 107°C) were higher than the non-chain-extended (101°C). This may be explained as the long-chain branched structures of chain-extended blends might inhibit crystallization of PLLA chains in the blends.

The T_g_ and T_m_ of chain-extended blends with different PLLA/copolyester ratios were similar in ranges 56–58°C and 173–174°C, respectively. The T_cc_ decreased from 104°C to 99°C and to 97°C when the copolyester ratios were 10 and 20 wt%, respectively. However the T_cc_ increased up to 108°C for the 40 wt% copolyester. This indicates that good compatibility between PLLA and copolyester could occur at lower copolyester ratios. Therefore copolyester phases could act as nucleating sites for PLLA crystallization.

The *X*_c_ of chain-extended PLLA (14.8%) increased up to 20.5% when 10 wt% copolyester was blended. The *X*_c_ of PLLA matrix decreased as the copolyester blend ratios were increased up to 20 wt% (*X*_c_ = 16.0%) and 40 wt% (*X*_c_ = 3.9%), indicating that low copolyester blend ratios (10 and 20 wt%) promoted the crystallization of PLLA. These results are attributed to the immiscible copolyester particles that might have acted as heterogeneous nucleating sites for crystallization of the PLLA matrix [29–31]. However, high copolyester blend ratio (40 wt%) inhibits the PLLA crystallization due to large copolyester particles (decreased interfacial area) as the copolyester blend ratio increased due to copolyester droplets coalescence during melt blending. It has been reported that the nucleation effect is directly related to the surface area of nucleating sites [31, 32].

### 3.2. Melt Flow Index

The MFI of the blends was used to assess their resistance of melt flow, as reported in Table 2. The MFI of pure PLLA in this work was 38.57 ± 1.75 g/10 min. The MFI of pure copolyester could not be measured as it was too liquid at 190°C. The MFI of 60/40 (w/w) blend without Joncryl® was 23.77 g/10 min which drastically decreased to 2.59 and 2.04 g/10 min for the 1.0 and 2.0 phr Joncryl® contents, respectively. Chain extension of PLLA/copolyester blends significantly decreased its MFI. This could be explained by the Joncryl®/polyester reaction which resulted in long-chain branched structures imparting resistance to melt flow characters [25]. Consequently, the copolyester could also react with the epoxide groups of Joncryl® molecules. Both PLLA and copolyester chains might be linked with the Joncryl® molecules. The MFI of chain-extended PLLA/copolyester blends decreased slightly as the copolyester blend ratio increased. Copolyester blending reduced MFI of the blends due to the entanglement of PLLA and copolyester chains [33].

### 3.3. Phase Morphology

Phase morphology has been widely used to explain the toughness of elastomer-toughened PLLA [31]. In this work, the compressed blend films were used to observe the phase separation between the PLLA matrix and dispersed copolyester droplets from their cryofractured surfaces.

**Figure 4:**
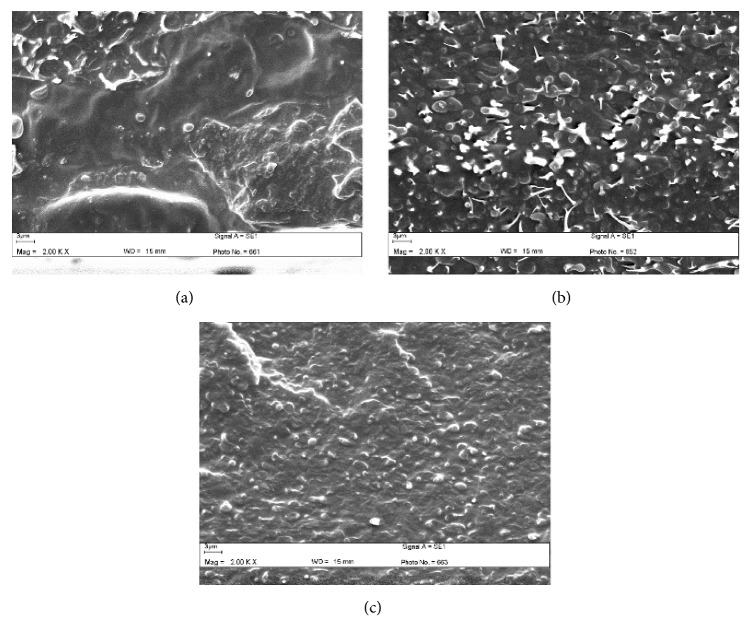
SEM images of cryofractured surfaces of 60/40 (w/w) PLLA/copolyester blends: (a) without and with Joncryl® contents of (b) 1.0 and (c) 2.0 phr (all bar scales = 3 *μ*m).

**Figure 5:**
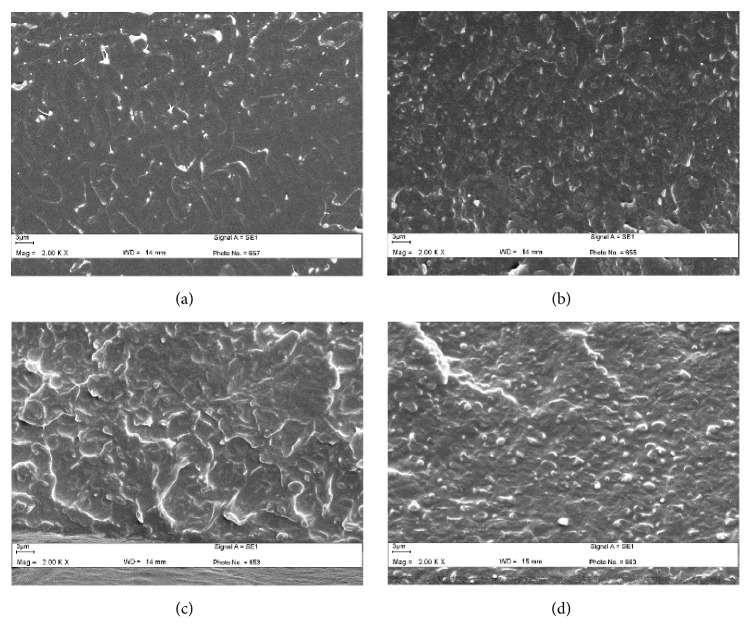
SEM images of cryofractured surfaces of chain-extended blends with PLLA/copolyester ratios of (a) 100/0, (b) 90/10, (c) 80/20, and (d) 60/40 (w/w) (all bar scales = 3 *μ*m).

The size of the dispersed copolyester droplets clearly decreased and distribution of copolyester droplets was improved when the Joncryl® content was increased as shown in Figure 4. The decrease in size of the dispersed copolyester droplets indicates compatibility between the PLLA matrix and the dispersed phase was then enhanced [30]. These results strongly indicate that the chain extension with Joncryl® enhanced phase compatibility between the PLLA matrix and dispersed copolyester phase. This may be explained by the fact that the addition of a chain extender in the PLLA/copolyester blends produced a few PLLA-copolyester block copolymers which could act as compatibilizers and enhanced the interfacial adhesion. In addition, no gap between PLLA matrix and copolyester droplets can be clearly observed for the non-chain-extended blend films in Figure 4(a), indicating that there was good phase adhesion. The lactide segments of copolyester chains also enhanced compatibility between PLLA and copolyester phases [16]. In addition, the stress-whitened copolyester particles of 60/40 (w/w) blend films in Figure 4(b) indicated a ductile fracture [31]. As shown in Figure 5, the copolyester droplet size of blend films increased with the copolyester blend ratio. This is due to the coalescence of the copolyester droplets during melt blending.

### 3.4. Tensile Properties

**Figure 6:**
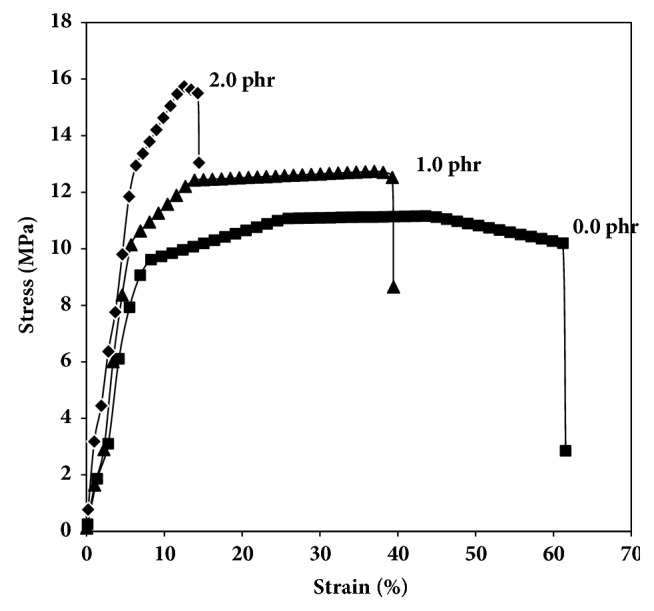
Tensile curves of 60/40 (w/w) PLLA/copolyester blend films prepared with various Joncryl® contents.

**Figure 7:**
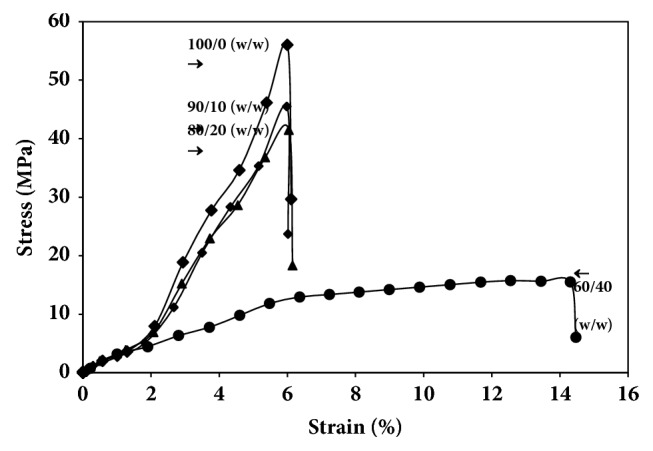
Tensile curves of chain-extended blend films prepared with various PLLA/copolyester ratios.

The tensile curves of the selected blend films as a function of Joncryl® content and PLLA/copolyester blend ratio are shown in Figures 6 and 7, respectively. The average tensile properties including stress at break, elongation at break, and Young's modulus are summarized in Table 3.

Young's modulus and stress at break of 60/40 (w/w) blend films slightly increased and elongation at break significantly decreased as the Joncryl® content increased, these results are similar to those reported by Ojijo and Ray [34]. The addition of Joncryl® had a minimum effect on Young's modulus and stress at break for the 60/40 (w/w) blend films. It would also appear that higher Joncryl® content might be leading to greater branching that reduced its elongation at break.

For the effect of PLLA/copolyester ratio, the chain-extended PLLA film is rigid and possesses low elongation at break (6.0%). All the chain-extended blend films with different PLLA/copolyester ratios exhibited lower Young's modulus and stress at break than the chain-extended PLLA film. Young's modulus and stress at break of the blend films slightly decreased as the copolyester blend ratio increased up to 20 wt% but the elongation at break did not change significantly. This is in line with literature, that is, the incorporation of an elastomer to the PLLA matrix decreases its Young's modulus and stress at break [31]. Young's modulus and stress at break of blend films drastically decreased and elongation at break greatly increased when the copolyester blend ratio was up to 40 wt%, due to the elastic nature of copolyester.

### 3.5. Impact Strength

The impact test was carried out by the notched izod method (resistance to crack propagation) and the impact strength results are also summarized in Table 3. Interestingly, the impact strength of the 60/40 (w/w) blends increased drastically from 4.31 to 14.5 and 16.9 kJ/m^2^ for the 1.0 and 2.0 phr Joncryl®, respectively. This indicates that the chain extension enhanced toughness of the blends. The morphological features of the dispersed rubbery phases greatly affected the toughness of PLLA blends [16]. Chain extension reaction improved phase compatibility of the blends to decrease the size of copolyester droplets as described above in the phase morphology section. The good phase compatibility between PLLA matrix and dispersed rubber phase enhanced the impact strength of the PLLA matrix [35]. This is in accordance with published work which reported that the formation of long-chain branched structures from the chain extension reaction improved the notched impact strength [36].

The chain-extended PLLA with brittle character (2.2 kJ/m^2^) showed lower impact strength than the chain-extended PLLA/copolyester blends (3.3–16.9 kJ/m^2^) due to the elastomeric nature of copolyester component which is able to absorb impact stress. The impact strength of chain-extended blends containing 2.0 phr Joncryl® significantly increased with increasing copolyester blend ratio. The results demonstrate that the dispersed rubbery droplets acted as stress concentrators during impact deformation [29] and improvement in toughness of PLLA matrix was then obtained.

### 3.6. Hardness

The hardness test was measured using shore D type. The effect of Joncryl® content and copolyester blend ratio on hardness of PLLA-based blends are reported in Table 3. The hardness of all the blends (57.4–81.2) were lower than that of chain-extended PLLA (87.2) for the 2.0 phr Joncryl® content. All blends were softer than the chain-extended PLLA, due to elastic deformation of the rubbery copolyester. The hardness of 60/40 (w/w) blends slightly increased with the Joncryl® content. This indicates that the chain extension reaction of blends induced stiffness. The hardness decreased steadily as the copolyester ratio in the blend increased.

## 4. Conclusions

Toughened and softened PLLA were successfully prepared by melt blending with a rubbery P(CL-*co*-LLA) copolyester in the presence of a chain extender. The chain extension reaction greatly suppressed crystallization of the PLLA matrix and greatly reduced the melt flow property of the blends. However, the crystallization of the PLLA matrix was improved for the 90/10 and 80/20 (w/w) blends but not for the 60/40 (w/w) blends. The morphology study of the PLLA/copolyester blends by SEM showed that PLLA and copolyester were immiscible but had good phase adhesion. The sizes of the dispersed copolyester droplets decreased as the Joncryl® content increased. The chain extension of PLLA/copolyester blends with Joncryl® improved the phase compatibility of PLLA-based blends. The presence of 40 wt% copolyester in the PLLA-based blends with and without chain extension improved the elongation at break and notched izod impact strength compared with the chain-extended PLLA. The copolyester blending reduced significantly the hardness of the PLLA matrix. In summary, chain extension and P(CL-*co*-LLA) copolyester blending showed synergistic effects to enhancing the melt flow property and toughness of the PLLA, thereby expanding the biomedical applications of PLLA.

## Figures and Tables

**Table 1:** DSC results of PLLA/copolyester blends.

PLLA/copolyester ratio (w/w)	Joncryl® content (phr)	T_g_ (°C)	T_cc_ (°C)	ΔH_cc_ (J/g)	T_m_ (°C)	ΔH_m_ (J/g)	*X*_c_ (%)
60/40	-	59	101	-15.8	175	29.8	25.1
60/40	1.0	57	108	-18.0	174	19.8	3.2
60/40	2.0	60	107	-18.1	175	20.3	3.9
100/0	2.0	58	104	-25.9	174	39.7	14.8
90/10	2.0	58	99	-23.1	174	40.3	20.5
80/20	2.0	56	97	-19.8	173	31.7	16.0

**Table 2:** MFI of PLLA/copolyester blends (190°C-2.16 kg).

PLLA/copolyester ratio (w/w)	Joncryl® content (phr)	MFI (g/10 min)
60/40	-	23.77 ± 0.20
60/40	1.0	2.59 ± 0.02
60/40	2.0	2.04 ± 0.05
100/0	2.0	4.80 ± 0.03
90/10	2.0	3.90 ± 0.02
80/20	2.0	3.84 ± 0.20

**Table 3:** Mechanical properties of PLLA/copolyester blends.

PLLA/copolyester ratio (w/w)	Joncryl® content (phr)	Young's modulus (MPa)	Stress at break (MPa)	Elongation at break (%)	Impact strength (kJ/m^2^)	Hardness Shore D
60/40	-	209 ± 58	10.9 ± 1.9	57.3 ± 8.2	4.3 ± 0.8	54.3 ± 1.2
60/40	1.0	240 ± 69	11.2 ± 2.2	40.0 ± 1.8	14.5 ± 0.6	57.4 ± 0.7
60/40	2.0	328 ± 16	14.6 ± 1.2	15.8 ± 6.0	16.9 ± 1.4	58.5 ± 0.9
100/0	2.0	1491 ± 38	57.6 ± 2.3	6.0 ± 0.3	2.2 ± 0.5	87.2 ± 0.8
90/10	2.0	1210 ± 101	44.8 ± 2.0	6.2 ± 0.2	3.3 ± 0.6	81.2 ± 1.8
80/20	2.0	1122 ± 46	41.8 ± 0.2	6.1 ± 0.1	5.6 ± 0.9	66.7 ± 0.8

## Data Availability

The data are clearly reported in the text and the article is fully consistent without the support of any additional data.
